# Leukemia and Benzene 

**DOI:** 10.3390/ijerph9082875

**Published:** 2012-08-14

**Authors:** Robert Snyder

**Affiliations:** Department of Pharmacology and Toxicology, Ernest Mario School of Pharmacy, Environmental and Occupational Health Sciences Institute, Rutgers the State University of New Jersey, Piscataway, NJ 08854, USA; Email: rsnyder@eohsi.rutgers.edu; Tel.: +1-732-445-5229; Fax: +1-732-445-5767

**Keywords:** benzene, bone marrow, niche, leukemia, benzene metabolism, stem cells, cell signaling, signal transduction, cytokines, cancer stem cells

## Abstract

Excessive exposure to benzene has been known for more than a century to damage the bone marrow resulting in decreases in the numbers of circulating blood cells, and ultimately, aplastic anemia. Of more recent vintage has been the appreciation that an alternative outcome of benzene exposure has been the development of one or more types of leukemia. While many investigators agree that the array of toxic metabolites, generated in the liver or in the bone marrow, can lead to traumatic bone marrow injury, the more subtle mechanisms leading to leukemia have yet to be critically dissected. This problem appears to have more general interest because of the recognition that so-called “second cancer” that results from prior treatment with alkylating agents to yield tumor remissions, often results in a type of leukemia reminiscent of benzene-induced leukemia. Furthermore, there is a growing literature attempting to characterize the fine structure of the marrow and the identification of so called “niches” that house a variety of stem cells and other types of cells. Some of these “niches” may harbor cells capable of initiating leukemias. The control of stem cell differentiation and proliferation via both inter- and intra-cellular signaling will ultimately determine the fate of these transformed stem cells. The ability of these cells to avoid checkpoints that would prevent them from contributing to the leukemogenic response is an additional area for study. Much of the study of benzene-induced bone marrow damage has concentrated on determining which of the benzene metabolites lead to leukemogenesis. The emphasis now should be directed to understanding how benzene metabolites alter bone marrow cell biology.

## 1. Introduction

Benzene, the commercial use of which dates to the late nineteenth century, was one of the earliest industrial chemicals demonstrated to affect the health of large numbers of workers [1,2]. Exposure to benzene varied from low concentrations, thought to be without effect in humans, to high concentrations, ranging up to many hundreds of parts per million (ppm), which resulted in workers displaying decreases in the numbers of erythrocytes, leucocytes and/or thrombocytes in circulating blood. Decreased numbers of all three cell types was termed pancytopenia and was usually found to be the result of aplastic anemia. Thus, it was agreed that the bone marrow was the target organ for benzene toxicity. We now recognize that benzene exposure can also lead to the development of myelodysplasia, a pre-leukemic state, and to one of the many types of blood cancers termed the leukemias.

For more than a century our main concern regarding benzene and human health has been the danger from industrial exposure to benzene. In the 21st century, as living standards in many countries around the world has risen and more people drive gasoline-fueled cars, the number of people who are exposed to benzene has expanded dramatically. Both benzene and gasoline are among the products derived from petroleum. Initially they are collected separately but later small amounts of benzene are added back to gasoline to prevent engine knocking. Thus, people may be exposed to fugitive emissions of gasoline vapor at the filling station, in the car and in garages attached to homes. To be sure, emphasis has been placed on protecting the public against exposure to benzene in the USA and Europe, but recent studies in China suggest that industrial exposure requires further attention. An expanded market for automobiles, especially in China and India, raises concerns. Furthermore, we have little information on exposure to benzene in gasoline, cigarette smoke or other potential sources in these densely populated countries. 

## 2. Current Scientific Issues in the Study of Benzene Leukemogenesis

Mechanistic toxicologists are of the opinion that we can make more rapid progress toward understanding the relationship between benzene exposure and benzene-induced leukemia if we can decipher the biochemical mechanisms that impel leukemogenesis [3]. In this discussion we plan to provide a brief synopsis of the historical development of our knowledge of benzene toxicity but will emphasize benzene-induced leukemia. We have been aware of the toxic effects of benzene for more than a century [4] but have lacked the knowledge and the tools necessary to effectively study the mechanisms by which benzene causes bone marrow malfunction. Although bone marrow was recognized to be the principal hematopoietic organ in adults, little was known about the internal morphology of the bone marrow and how the arrangement of the various components influenced bone marrow function. Recent studies have provided an illuminated view of the inner workings of the bone marrow [5,6]. It is now clear that the marrow is a highly structured organ with various processes occurring in areas called “niches”, each of which appears to be located at predetermined locations with respect to the bone and the circulating blood. Furthermore, there have been dramatic advances in our understanding of the humoral control of the bone marrow processes termed proliferation and differentiation [7]. If we are to fully understand the underlying basis of benzene-induced bone marrow function we must explore the role played by regulatory mechanisms such as signaling pathways that appear to direct some aspects of the mechanism of benzene-induced leukemia. Both of these areas will be discussed below and some suggestions on how a better understanding of bone marrow function can help to definitively describe the mechanism of benzene-induced leukemia will be proposed.

## 3. Benzene Production

To understand the magnitude of the benzene problem in the modern world it would be useful to present a brief summary of how benzene production has grown since its discovery. People have been exposed to benzene since earliest times as a result of its release during the burning of wood, tobacco, and other carbon-containing materials. Its industrial use became significant as a byproduct of the production of illuminating gas early in the 19th century [8]. The gases were released upon the destructive distillation of whale or cod oil by heat. The gases were then compressed and stored in portable vessels that were distributed to customers for use in their homes or workplaces. It was observed that the compressed gases released an oil on standing. Michael Faraday isolated benzene from the condensed oil in 1825 and gave it the name “carburet of hydrogen”. He determined that its chemical composition was C_6_H_6_, but it was not until 1865 that Kekulé proposed the ring structure that is accepted today. 

Coke is a pure form of carbon derived by heating coal to high temperatures anaerobically in coke ovens. These can be designed to collect many of the volatile components of coal, including benzene (2 kg benzene for 3 kg volatile organics) [9] and also from a complex mix of chemicals which are melted out of the coal, referred to as coal tar. Hofmann demonstrated in 1845 that he could isolate benzene from coal tar [10]. Riegel suggested in 1949 that 1 ton of coal could produce about 10 gallons of tar, and 2 gallons of benzene [11]. Batchelder (Battelle Memorial Institute, Columbus, OH, USA) in a report entitled “*Chemicals from Coal*” reviewed the field up to 1962 [12]. In 1910, 70 million tons of coke was produced in the United States without the recovery of byproduct chemicals, in part because at that time it was possible to purchase benzene from Germany. With the onset of WWI byproduct coke ovens were installed and coking capacity increased to 97 million tons and remained level through the mid 1950s. The annual production of benzene from coking also remained level at about 150–200 million gallons during 1940–1955. 

Isolation of benzene from petroleum began in the early 1940’s and by 1950 about 10 million gallons of benzene per year were derived from petroleum. The increase in production was then quite steep and surpassed the coking procedure to yield about 600 million gallons by the early 1960’s. C & E News surveyed the production of major commodity chemicals during the period 1989–1998 [13]. During that time the average production of benzene from petroleum was approximately 1,630 million gallons. 

Given the amount of benzene produced, and its widespread use, it is difficult to find people who have not experienced benzene exposure during their life time. There is no reason to expect that the demand for benzene will subside in the coming years. Despite precautions taken by governmental and industrial organizations to protect people from exposure to benzene, as more benzene is produced the potential for human exposure continues to increase.

## 4. Protection against Benzene Exposure

The approach used in many nations to protect workers from excessive exposure to chemicals has been to explore the dose-response relationship linking airborne concentrations of the chemical to adverse responses. In theory, the age old admonition of Paracelsus, *i.e.*, the dose determines whether or not the chemical will act as a poison, suggests that there may be exposure levels below which the adverse effect under study may not be observed. Toxic effects such as depression of the production of blood cells as a result of chronic exposure to benzene are thought to be threshold driven, *i.e*., there is some level of exposure below which it would be unlikely to observe toxic effects such as decreases in circulating blood cells.

The U.S.E.P.A. in a document entitled *Carcinogenic Effects of Benzene: An Update* (April, 1998) states that “Currently there is insufficient evidence to deviate from using an assumption of a linear dose-response curve for benzene, hence, the Agency’s past approach of using a model with lowdose (sic) linearity is still recommended [14]”. That suggests that a specific risk can be associated with each dose at extremely low doses. Attempts to find acceptable levels of benzene exposure as a result of either studies of human epidemiology or animal bioassays have failed to provide sufficient evidence to counter the regulatory decision. Nevertheless, as a practical matter the U.S. Occupational Safety and Health Administration has set Permissible Exposure Limits (PELs) for industrial exposure to benzene. OSHA has established that “*The employer shall assure that no employee is exposed to an airborne concentration of benzene in excess of one part per million parts of air (1 ppm) as an 8-hour time-weighted average*”. Furthermore, “*The employer shall assure that no employee is exposed to an airborne concentration of benzene in excess of five (5) ppm as averaged over any 15 minute period*”. 

The United States Environmental Protection Agency (USEPA) evaluates the potential danger of exposure to chemicals in the environment and publishes the risks of exposure in its Integrated Risk Information System (IRIS). It has established a reference dose for the inhalation of benzene (Rfc): “*the RfC is an estimate (with uncertainty spanning perhaps an order of magnitude) of a daily inhalation exposure of the human population (including sensitive subgroups) that is likely to be without an appreciable risk of deleterious effects during a lifetime*”. According to the IRIS document (available on line) exposure in the range of 13.0 to 45.0 µg/m^3^ (4.07–14.11 ppm) could yield 1 cancer in 10,000 exposed people. Reducing the exposure by 10 or 100 fold would reduce the risk 10 or 100 fold, respectively. 

## 5. Background

Occupational Medicine and Toxicology are two closely interrelated disciplines. The recognition that people working in specific occupations, e.g., miners, often showed signs of similar illnesses appeared early in the scientific literature [2]. Ramazzini made it clear in 1713 that it was quite common for workers to be harmed as a result of exposure to the materials with which they were working [15]. However, prior to the industrial revolution of the 18th and 19th centuries there were few, if any, work places where large numbers of workers were exposed to the same materials and formed a recognizable cohort that might attract the attention of observant medical practitioners. The observed effects on individual workers were not recognized to be anything other than diseases that naturally occurred in the general population. Unfortunately, no steps were taken to protect the workers, and if they became ill or died they were simply replaced. Furthermore, the science of Toxicology, and of the overarching sciences of Biology and Chemistry, were not sufficiently advanced to permit exploration of the specific chemicals and their mechanisms of action to enable medical intervention to protect or treat impaired workers.

Uglow described the growth of large scale industry in Britain in the 18th century largely as a result of scientific and technical advances [16]. For example, the development of the steam engine by James Watt fostered the emergence of the weaving industry in large factories that employed many workers using automated weaving machines to manufacture cloth. In many cases the success of these industries was closely tied to the utilization of newly discovered chemicals and new processes [2]. Under these circumstances it became clear that exposure to specific chemicals could result in similar illnesses in a significant number of individuals employed in these modern facilities. 

Benzene is an example of a chemical to which few people were exposed in large amounts prior to its development as a solvent in the rubber industry and later as a solvent for inks, paints and other water-insoluble materials. The first report linking benzene exposure to any disease was presented at the 12th International Congress of Medicine by Dr. C.C. Santesson, Professor of Pharmacology at the University of Stockholm, in 1897. He reported on nine women, four of whom died, who worked in a rubber tire factory in Uppsala. They displayed *purpura húmorrhagica* (an impairment of blood clotting); today we would classify their condition as aplastic anemia. They were exposed daily to benzene which was used as a solvent for the rubber. Santesson concluded that benzene was “…*das wesentlich toxische Princip*…”, *i.e*., the principle agent that caused their disease. Several similar cases were seen at Johns Hopkins Hospital by Selling [17]. He went on to demonstrate that injection of benzene into rabbits produced similar effects [18]. Weiskotten [19] exposed rabbits to benzene via inhalation (the route by which most industrial intoxications occur) to 240 ppm of benzene (see [1]) and confirmed these results. Since then there have been reports of workers exposed to benzene in a variety of industries with observations including decreased levels of circulating blood cells of varying severity, the most serious of which is aplastic anemia (e.g., [20,21,22]).

The discipline of Occupational Medicine became a significant medical specialty because of physicians like Alice Hamilton, who was an early proponent of the concept that limiting exposure of workers to chemicals would reduce the incidence of occupational diseases [23]. However, as Toxicology matured as a discipline it became clear that it was necessary to examine the relationship between the degree of exposure to these chemicals and the adverse effects that they produced. Furthermore, rapid advances in our appreciation of Physiology and Pathophysiology during the last century have driven Toxicologists to explore the biochemical mechanisms that shape the development of these diseases. For approximately a half century after the discovery of benzene toxicity the literature focused on descriptive studies in humans and animals demonstrating, for the most part, circumstances leading to benzene-induced decreases in circulating blood cells. 

## 6. Leukemia

Throughout history physicians, when confronted with a diseased patient, have attempted to identify the disease based on examination of the patient and references to the medical literature. The development of diagnostic procedures over the ages has been limited by our lack of basic information on human anatomy, physiology and pathology. Furthermore modern diagnostic techniques rely heavily on technological advances that have been products of the last few centuries. During that time many diseases were “discovered” and described by physicians who were acute observers and who communicated their observations to the medical community. Freireich and Lemak reported in 1991 that Alfred Velpeau, a French surgeon, described the case of a man presented at a hospital in 1827 with a swollen abdomen, fever, and weakness [24]. He soon died and at autopsy was shown to have an enlarged liver and spleen, and thick “gruel-like” blood. Velpeau asked a critical question: “*Does the condition of the spleen and liver cause the decomposition of the blood, or, could it be that the abnormal fluid produces enlargement of these organs?*”

In 1837 Alfred Donné established a course in clinical microscopy within the medical faculty at the University of Paris. He was aware of the presence in blood of three types of cells, *i.e*., red cells, white cells (also termed mucous globules) and platelets, termed “small globules”. He was sent a sample of blood from a patient, who in retrospect was probably leukemic, to evaluate and determined that more than half of the cells were mucous globules. He stated that: “This fact needs perhaps some explanation”. There were questions raised because these samples were obtained after the death of the patient. In 1844, however, Donné reported having made similar observations in several blood samples taken from live patients. Donné may be credited with the first report describing leukemia as a hematological abnormality.

In 1845 two papers appeared that generated a long lasting dispute concerning the reason for the elevated white blood cells in these diseases. In the first, John Hughes Bennet, a Scottish physician who had studied with Donné, suggested that the blood contained large amounts of pus, *i.e*., it was suppurated [24,25]. He thought that the pus arose from an infection, but upon autopsy could find no evidence of an infection. He suggested that this was a spontaneous generation of pus in the blood. Rudolph Virchow, Professor of Pathology at the University of Wurzburg, suggested that this was a disease of the white blood cells and he invented the term “leukämie”, *i.e.*, leukemia. The recognition that leukemia was a true disease entity was enforced by the work of Julius Vogel, Professor of Medicine, University of Giessen, who in 1854 reported observations on 25 patients. He described leukemia as a chronic disease that developed slowly but eventually led to death. Nickolaus Friedreich, the successor of Virchow in Wurtzburg, reported the first case of acute leukemia in 1857. Ernst Neumann, a student of Virchow and Professor of Pathology at the University of Königsberg, was a pioneer in the field of hematology. He demonstrated that blood cells arose from the bone marrow, and that leukemia was a disease of the bone marrow. He coined the term myelogenous leukemia. 

Since then major efforts have been devoted to the classification of different forms of hematopoietic neoplastic diseases. The major distinction between these has been that some leukemias develop slowly over months and years leading to high levels of circulating white blood cells, and are termed chronic leukemias. Acute leukemias are characterized by early overgrowth of white cells that are frequently immature and the disease is usually fatal within a short time. Although advances have been made in treating the leukemias, in many instances they continue to be fatal. 

A review of the World Health Organization classification of leukemias was presented in 2010 by Dr. James Vardiman at the symposium entitled “*BENZENE 2009-Health effects and mechanisms of bone marrow toxicity: implications for t-AML and the mode of action framework*” [26]. The document cited 22 types of hematopoietic and lymphoid neoplasms and subdivided these into approximately 145 subcategories. Data regarding the frequency of any of these diseases will be dependent upon the physician and the technical capabilities of the laboratory assisting in the diagnosis. 

## 7. Benzene Exposure and Leukemia

The suggestion that benzene exposure could result in leukemia was more difficult to establish than the demonstration that benzene could induce aplastic anemia. Benzene-induced decreases in blood cells could be observed within a few months after exposure was initiated. However, there is a lag time of perhaps years between initial benzene exposure and the development of leukemia. Early reports associating benzene exposure with leukemia represented individual cases [27,28,29]. Penati and Vigliani of the Institute for Industrial Medicine, University of Turin, reported in 1938, the first estimates of the frequency of leukemia among workers exposed to benzene at their jobs during 1928–1938 [30]. They found 60 cases of aplastic anemia and 10 cases of leukemia. Following WWII Vigliani and coworkers at the University of Milan surveyed workers exposed to benzene during 1942–1975. In Milan 66 cases of hemopathy were observed leading to death caused by aplastic anemia in seven cases and 11 of leukemia. In a separate study in the city of Pavia they found 137 cases of hemopathy leading to death from aplastic anemia in three cases and leukemia in 13 cases [31]. During the period 1955–1960 the shoe making industry in Istambul, Turkey, switched from using petroleum-based glues to one that contained benzene. Aksoy and co-workers (1974) at the University of Istambul found that with the change in solvents they began to see large numbers of people with hemopathies including 26 leukemias among approximately 28,500 shoe makers [32]. 

In the mid-1970’s the United States Occupational Health and Safety Administration (OSHA) planned to institute a new permissible occupational exposure limit for benzene and held administrative law hearings intended to develop an argument to support changing the standard. Although the strongest evidence supporting the idea that benzene was a leukemogen came from the work of Vigliani and Aksoy (Aksoy testified at these hearings), OSHA sponsored a new study of benzene-exposed workers in the rubber industry, to demonstrate whether or not these effects could be observed in American Workers. Their efforts were rewarded when Infante *et al.* reported a significant increase in myeloid leukemias in benzene-exposed workers at a rubber plant in Akron, Ohio [33]. 

Since that time others have examined the relationship of benzene exposure to the development of leukemias, but the studies that have attracted most attention have been those of Yin, Li, and their Chinese coworkers and their colleagues at the U.S. National Cancer Institute. In an early report, they described a study in which a cohort of 35,805 control subjects was compared with 74,497 benzene-exposed workers [34]. Analysis of the data revealed that there were significant increases in the benzene-exposed group in acute myelogenous leukemia, malignant lymphoma, myelodysplastic syndrome and aplastic anemia. These and subsequent studies supported the concept that benzene was a leukemogen. 

## 8. Benzene Metabolism

Xenobiotic metabolism usually modifies chemicals to make them more water-soluble by introducing oxygen or other polar groups into the potentially dangerous chemical. The process was termed “detoxication” by R.T. Williams [35] but unfortunately metabolic transformation by the xenobiotic metabolism system often leads to more toxic metabolites. Thus, Parke and Williams provided an early description of the pathway of benzene metabolism based on the administration of radiolabeled benzene to rabbits and suggested that benzene toxicity might be due to the production of toxic metabolites [36,37].

**Figure 1:**
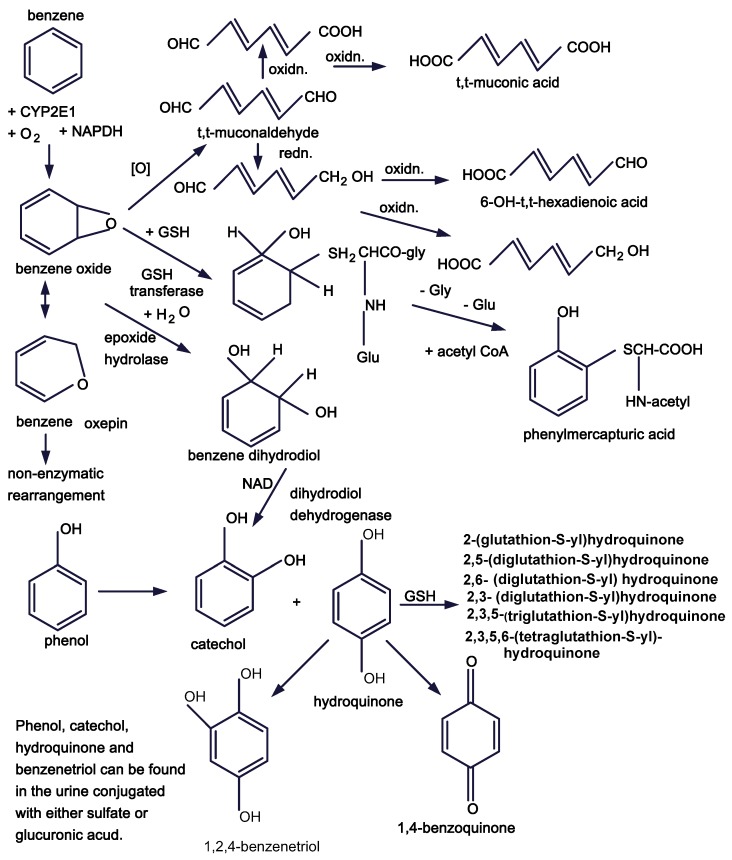
Benzene metabolic pathways.

The metabolism of benzene, as described by Snyder and Hedli, begins in the liver with the introduction of oxygen via cytochrome P4502E1 to yield benzene oxide, which can then spontaneously rearrange to phenol (Figure 1) [38]. Benzene oxide can also be acted upon by epoxide hydrolase to yield benzene dihydrodiol which may be enzymatically converted to catechol, or it can be conjugated with glutathione and eventually excreted as phenylmercapturic acid. The benzene oxide ring may also be opened to form a series of six carbon dienes, the most reactive of which is *tt*-muconaldehyde and the excretory form of which is *tt*-muconic acid. Phenol can also be hydroxylated to form catechol, hydroquinone and 1,2,4-trihydroxybenzene. Hydroquinone can be oxidized to 1,4-benzoquinone. Phenol and all of its metabolites can be excreted as either sulfate or glucuronide conjugates. The most recently examined benzene metabolites thought to have biological activity include a series of hydroquinone conjugates with glutathione, including GSH binding to positions 2, 2 and 5, 2 and 6, 2 and 3, 2, 3, and 5, and 2, 3 and 6 of the hydroquinone ring [39]. The general property of the GSH moieties bound to the hydroquinone ring is that they promote oxidative cycling of ring hydroxyl groups leading to the production of reactive oxygen species. Among these metabolites the most potent in inhibiting erythropoiesis are *tt*-muconaldehyde and 1,4-benzoquinone. Less potent but also effective are hydroquinone and catechol, co-administration of phenol with hydroquinone and catechol, and some glutathione adducts of hydroquinone. Despite intensive efforts in many laboratories, given the structural variety and the significant chemical reactivity of these benzene metabolites it has been difficult to determine which may contribute most to producing benzene toxicity or leukemia. 

## 9. The Target Organ: The Hematopoietic System of Bone Marrow

Hematopoiesis is a complex process that occurs in the bone marrow and insures that the proper number and type of circulating blood cells can be produced during one’s lifetime [40]. Normally the levels of effective blood cells in the circulation are maintained by a system in the bone marrow, that is comprised of hematopoietic stem cells (HSCs) and their progeny. HSCs have been termed pluripotential because they can give rise to erythrocytes, each of the leucocyte lineages, and platelets via directed differentiation. These processes are controlled by a variety of factors including cytokines, and growth factors of various types that are released from cells within the marrow or from other sites within the body, e.g., erythropoietin, which stimulates red cell production is a product of the kidney. The stromal cells of the bone marrow create a microenvironment that supports the differentiation and proliferation of precursor cells derived from stem cells and eventually formed the mature cells of the circulation.

During the course of differentiation leading toward full maturation, precursor cells undergo proliferation to insure that sufficient numbers of blood cells are produced. On a microscope slide a smear of a bone marrow sample contains many precursor cells at later stages of differentiation, with few HSCs and cells at early stages of differentiation. There will be more cells at later stages of differentiation because of proliferation. The slide will also contain stromal cells, mature cells and other cells which may not be involved in hematopoiesis. 

A number of technical impediments had to be overcome before it was possible to understand the interaction of stem cells, progenitors, and stromal cells in the marrow. In a series of seminal papers Till and McCulloch and their coworkers demonstrated that upon injection of bone marrow cells from donor mice intravenously into mice that had been irradiated to inactivate their bone marrows, a significant number of cells from the donor mice migrated to the spleens of the recipient mice [41,42,43]. Subsequent inspection of the spleens revealed surface nodules, that when examined were determined to contains colonies of cells arising from a single cell. As a result they were termed “colony forming units” or CFUs. 

The cells within the colonies included additional CFUs as well as more differentiated progenitor cells suggesting that the CFUs were stem cells capable of self-renewal as well as differentiation. In several laboratories it has been shown that when benzene was administered by injection or by inhalation to donor mice there was a decrease in spleen colony-forming units [44,45,46,47]. 

The analysis of bone marrow function and the relationship between the cells progressed rapidly with the development of techniques to clone bone marrow cells using specific cytokines and other growth factors [48,49,50]. The procedure involves cloning cells found at specific stages in its development and stimulating them to differentiate and/or proliferate by applying appropriate growth factors. The number of cells that were successfully cloned can be estimated by counting the number of visible colonies that result. The U.S.E.P.A. reviewed in 2002 many studies in which the effects of adding benzene metabolites to cultures of colony forming units resulted in inhibition of the growth of the colonies [51]. A number of cell types have been cloned and the assays were used to detect effects of benzene. For example, benzene treatment resulted in decreases in CFU-E, BFU-E, which are early stages in erythroid differentiation, and CFU-GM an early stage in granulocyte macrophage development [52]. 

## 10. Regulation of Hematopoiesis

The regulation of the hematopoietic process is primarily maintained by a series of cytokines plus other proteins via processes termed signaling. The development of our understanding of the cytokine signaling pathways in hematopoiesis and in leukemogenesis was reviewed by Rane and Reddy [53] and Smith and Griffin [54]. These proteins control the differentiating or proliferating activity of otherwise inactive HSCs and of HSPCs. The process by which extracellular signals can influence intracellular activity is called signal transduction. The process of signal transduction is directed to accepting an extracellular signal, such as a cytokine or growth factor, at cell surface receptors, and conveying that signal through the cell membrane to initiate any of a series of intracellular message cascades that stimulate DNA-directed transcription and, ultimately, the production of specific proteins. In the bone marrow those proteins will inform the hematopoietic mechanisms to fill the need for the production of specific circulating cells. 

There appear to be two primary pathways of signal transduction that control hematopoiesis: the Src family of protein tyrosine kinases (PTK) and the cytokine receptors of the JAK-STAT pathway. There are about a dozen different growth factors that can act as ligands for the PTKs. In contrast, the JAK-STAT pathways involve receptors that respond to cytokines, e.g., colony-stimulating factors and interleukins. 

The Src PTKs were discovered based on the observation of Rous in 1910 that a filterable particle derived from a chicken tumor could yield a similar tumor when injected into another chicken [55]. Eventually it was found that these particles were viruses and that their genetic machinery coded for protein kinases, that phosphorylated tyrosine sites on other proteins [56,57,58,59]. PTKs are a diverse group of trans-membrane enzymes in which the extracellular portion acts as a receptor for ligands such as epidermal growth factor, fibroblast growth factor, insulin and insulin-like growth factors, erythropoietin, *etc*. Normally monomeric, the binding of a ligand causes dimerization of polypeptide chains that results in phosphorylation on tyrosines of intracellular proteins. The mechanism linking extracellular ligand formation and intracellular kinase activity is unknown [60,61]. 

The cytokine receptors do not act as kinases but when stimulated by specific cytokines activate the JAKs which are a group of kinases that constitute the Janus family of kinases. Baker *et al.* suggested in 2007 that there were five families of cytokine receptors each of which responds to a specific cytokine [62]. They postulated that JAKs may be associated with the receptors but are inactive until the receptor is bound by a ligand. At that point dimerization occurs leading to activation of kinase activity by phosphorylation of their tyrosine residues. The activated JAKs may now phosphorylate the receptor to create docking sites for a number of signaling molecules including STATs, other Src kinases, phosphatases, *etc*. Once the signal has passed into the cell membrane via either of these mechanisms there are a number of possible pathways by which the signal may reach the nucleus and impact on the DNA. 

## 11. Intracellular Signaling

The literature is profusely illustrated with colorful schemes showing the many intracellular signaling pathways that transmit the signals derived from the kinases (the activities of which are influenced by trans-membrane transduction) through the cell to directly or indirectly influence transcription at the level of DNA. Rather than review each of the postulated pathways and their impact, it would be instructive to examine the report of a recent study that attempted to examine the roles of several intracellular signaling pathways below the level of membrane signal transduction in bone marrow cells by adding inhibitors of each of several pathways and measuring their effects on the production of colony-forming units *in vitro*. 

Bugarski *et al.* examined some aspects of the inhibition of specific intracellular signaling pathways on differentiation and proliferation of bone marrow precursor cells [63]. They recognized that leukemias are usually characterized by the excessive growth of incompletely differentiated bone marrow precursor cells. The early stages of differentiation involve activation of HSCs leading to a bifurcation whereby one branch proceeds to differentiate along the lymphoid cell line and the other along the myeloid cell lines, *i.e*., granulocytes, erythrocytes, monocytes and megakaryocytes, and early forms of which can be studied as the CFU-GEMM, a polyfunctional stem cell. Their experiment was specifically to measure the impact of inhibition of specific signaling pathways, in mouse bone marrow, on the production of BFU-E, (an early erythroid precursor), CFU-E (a more highly differentiated erythroid precursor), and CFU-GM (a precursor to granulocytes and macrophages). 

They asked the following question: If hematopoietic growth factors and cytokines activated by signal transduction at each of their receptors at the same time, thereby turning on a number of signaling cascades, how could this process lead to differentiation of specific precursors? The experiment that they designed was to determine what the effect would be on either erythroid or myeloid developing cells if they added inhibitors to each of several signaling pathways but allowed the remainder of the signaling pathways to proceed unchecked. 

They examined the roles of the PTKs, the mitogen-activated protein kinase (MAP), and the phosphoinositide-3-kinases (PI-3) signaling pathways on each of the three stages of differentiation in the myeloid or erythroid lineages. The cytokines used in the cultures, *i.e*., SCF (mouse stem cell factor), interleukins 3 and 6, and erythropoietin, activate several signal transduction pathways. The strategy used was to add inhibitors, which they considered to be specific for the signaling pathways to cell cultures and to then measure the development of the colony forming units. 

They found that inhibitors of PTKs such as genistein, which inhibits receptor tyrosine kinases, tyrphostin AG490, which inhibits Jak2 and Jak3 PTKs, and PP2, an inhibitor of SRC kinases, inhibited the growth of erythroid progenitors but not myeloid colonies. Among the MAP kinase inhibitors SB205380, which inhibits the p38 MAP kinase pathway, had no effect on CFU-GM but inhibited both BFU-E and CFU-E. SP600125, which inhibits JNK suppressed all progenitors [64]. PD98059, which acts on the MEK1/2-ERK1/2 pathway, had no effect on CFU-GM or CFU-E but decreased BFU-E. A study of PI-3 inhibitors showed that the selective inhibitor LY29403, the irreversible inhibitor Wortmannin, and the non-selective inhibitor quercetin suppressed the growth of all progenitors studied. The transcription factor NFkB suppressed all cultures. 

In 2002, the U.S.E.P.A. reviewed many studies in which the effects of adding benzene metabolites to cultures of colony forming units resulted in inhibition of the growth the colonies [51]. However, these studies did not attempt to explore the effects of the metabolites on signaling mechanisms. The studies described by Bugarski *et al.* suggest that it is possible to examine the role of inhibitors of various steps in differentiation and proliferation of bone marrow intermediates to provide indications of mechanisms by which inhibitors may alter hematopoiesis *in vivo* [63]. They make it clear that these are complex interactions and the results may reflect on more than a single signaling pathway. Furthermore, the inhibitors may impact on different stages of differentiation in more than a single lineage. The impact of the inhibitors may occur via an effect on receptors, enzymes, microenvironmental factors, *etc*. However, the use of a similar strategy to study the effects of benzene, that are largely the result of the generation of a variety of metabolites, provides an opportunity to propose any of several different challengeable hypotheses to evaluate a number of different potential mechanisms which could lead to hematotoxicity or leukemogenesis. 

## 12. Hematopoietic Stem Cells (HSCs)

A key feature of organs throughout the body is their accessible morphology that can be observed grossly, microscopically and even at a level beyond the capability of the eye if one uses an electron microscope. These techniques are possible because the organs can be viewed *in situ* or can be removed without loss of their structural characteristics even after dissection. HSCs arise in the bone marrow and were long thought be a homogeneous population of cells. Observation of the bone marrow has been limited because removal from the bone results in loss of any structural features that it may contain. Recent studies of bone marrow function *in vivo* and *in vitro* have led to the conclusion that bone marrow may be a highly structured organ. The unique structural features ascribed to the bone marrow are termed “niches”. 

Wilson and Trumpp [5] and Butler *et al*. [65] suggest that there are at least two types of HSC niche. One type may be a storage site for quiescent HSCs that are maintained in G_0_ and may undergo classical symmetrical mitosis, *i.e*., self renewal [66]. Another may be a niche in which HSCs undergo asymmetric mitosis, *i.e.*, to yield one HSC and a cell poised to begin the process of differentiation. Muller-Sieburg *et al.* suggest that there may be subpopulations of HSCs and have described three possible types of HSCs based on their differentiation capacity to follow the myeloid line, the lymphoid line, or a third type of cells that yield a balance of both forms [67]. The lymphoid type predominates early in life, and the myeloid type increases with age. Dykstra and de Haan discussed changes in HSCs that occur through aging [68]. As a result, we have age-related abatement of regenerative potential, reduced immune-competence, and the greater likelihood of myelogenous diseases such as myelodysplastic syndrome and leukemia. 

Rossi *et al.* reported that with age HSCs display decreased functional capacity in response to stress and accumulation of DNA damage [69]. The cells demonstrate impaired renewal and proliferative potential, increased apoptosis, and accumulation of DNA damage. Detection of DNA damage and the response are controlled by a signal transduction pathway that may result in DNA repair, alteration of the cell cycle or apoptosis. Critical to the fidelity of these responses are checkpoints that help to direct the response. Wang *et al*. described a differentiation checkpoint that limits hematopoietic stem cell renewal in response to DNA damage and also promotes tissue aging [70]. They identified a basic leucine zipper transcription factor (referred to as ATF-like, BATF) that is important in controlling self-renewal of HSCs in response to telomere dysfunction and γ-irradiation. In the absence of BATF HSC self-renewal was improved and the accumulation of DNA increased. When BATF was up-regulated less DNA damage was observed and differentiation was enhanced, thereby reducing the number of DNA damaged HSCs, perhaps due to the activation of the checkpoint proteins p53 and p21. 

Wang *et al*. concluded that these and other data suggest that decreased activity of the G-CSF/STAT3/BATF extends self-renewal despite DNA damage and permits continued accumulation of DNA damage in quiescent HSCs [70]. Differentiation itself is a checkpoint that prevents survival of damaged HSCs that in turn could evolve into “cancer stem cells”. The authors noted that HSCs enter the cell cycle only once every 3–4 months [71]. Aberrant DNA repair can occur in quiescent stem cells despite cell cycle arrest [72]. Quiescent cells are resistant to apoptosis stimulated by DNA damage and more likely to survive despite DNA damage [73]. Thus, differentiation may eliminate accumulation of genotoxic damage in quiescent HSCs and protect against subsequent development of DNA-associated damage leading to cancer. 

## 13. Cancer Stem Cells

Whether or not cancers arise from stem cells has been a matter of some debate [74,75]. Prevailing theories of carcinogenesis suggest that attack on DNA by a reactive metabolite(s) of a chemical, or by reactive oxygen generated during the metabolism of the chemical, results in either covalent binding of the metabolite(s) to DNA or oxidative damage caused by reactive oxygen species. Failure to repair DNA from such attacks may cause carcinogenic mutations and the generation of cells that can give rise to various forms of cancer. One can argue that if these cells undergo self-renewal, and if their progeny can differentiate (albeit in limited fashion) and proliferate, they might be termed cancer stem cells. The concept of the cancer stem cell provides a useful basis for this discussion. 

Bonnet and Dick (1997) reported finding a cell “*capable of initiating human AML*” when studying non-obese diabetic immune deficient mice (NOD/SCID mice) [76]. These cells displayed the capacity to differentiate and proliferate, as well as to self-renew. The cell surface of both these and normal SCID-repopulating cells were characterized by the surface markers CD34++ and CD38−. The author concluded that these were likely AML stem cells and arose from a leukemic transformation of normal SCID stem cells. 

Chemical carcinogenesis appears to provide a satisfactory explanation of tumors induced by many chemicals such as aflatoxin [77], benzo[a]pyrene [78], and others where high levels of DNA adduct formation are observed. In the case of benzene, and its role in leukemogenesis, DNA adducts have been described [79] but little DNA binding has been observed *in vivo* in animal test species [80]. Alternatively, it has been suggested that the benzene metabolite 1,4-benzoquinone may inhibit topoisomerase II and, thereby, inhibit the annealing of strand breaks in DNA resulting in mutations [81,82,83]. The use of topoisomerase II inhibitors as cancer chemotherapeutic agents often results in leukemia following remission of the earlier tumor. Topoisomerase II inhibition may play a role in the induction of benzene-induced leukemia. Another relatively unexplored potential impact of benzene metabolites that may impact on leukemogenesis is extensive covalent binding to proteins [84]. The many signaling pathways described above may be subject to attack by reactive metabolites of benzene leading to inactivation of critical signaling proteins or their receptors. 

The discussion by Wilson and Trumpp and their description of how niches may function in hematopoiesis plus the discussion of HSCs above suggests possible mechanisms which may be involved in the generation of benzene-induced leukemias [5]. One may visualize normal hematopoiesis involving two types of niches. In one (Type A) HSCs undergo asymmetric mitosis as needed generating a HSC and a cell poised to enter the differentiation scheme leading to mature circulating blood cells. In another (Type B) quiescent stem cells (HSCQs) are stored and await recruitment. Upon recruitment their numbers may be restored via self-renewal by symmetric mitosis. 

From time to time trauma, disease or DNA damage via reactive metabolites of environmental chemicals or reactive oxygen species produced during their metabolism could lead to cell death and a reduction in the functional residual capacity of the Type A niche to replace lost cells. It should be noted that exposure to benzene is likely to lead to bone marrow cell death and DNA damage at any stage of life. Chronic exposure would exacerbate these effects. Indeed people chronically exposed to benzene demonstrate chromosome aberrations prior to developing leukemia. 

Depletion of HSCs could lead to activation of HSCQs and their translocation to the Type A niche to restore homeostatic balance to hematopoiesis. HSCQs could then undergo self-renewal to maintain the proper number of stored cells. With age DNA repair mechanisms and the apoptotic response to DNA damage tends to regress. DNA damage in HSCQs may accumulate over time and eventually give rise to mutated cells that might be leukemia stem cells (HSCL). These cells would have different characteristics than HSCs or HSCQs and could reside in a different niche (Type C) and from there generate leukemia. 

It is noteworthy that there are reports suggesting that the time from initial exposure to benzene and subsequent development of leukemia may be anywhere from 5 to 20 years. Over time reduced capacity of mechanisms such as DNA repair, apoptosis, or effective checkpoints, or other genetic differences may have determined the time needed for leukemogenesis. 

## 14. Conclusions and Future Directions

The aim of this discussion has been to reprise some aspects of the interaction between benzene and the people whose lives it may impact. The review is somewhat incomplete because time and space do not permit a more exhaustive presentation the literature on benzene. However, there has been an attempt to suggest that there are some areas that require further evaluation.

- It is important that we begin to examine the impact of benzene metabolites and/or reactive oxygen species formed during benzene metabolism on signal transduction pathways and on cytokine receptors. Direct effects of reactive metabolites, e.g., *tt*-muconaldehyde, 1,4-benzoquinone, reactive oxygen species, *etc*., may interfere with signaling pathways.
- The factors involved in conversion of a normal cell to a leukemia cell, that may be termed a leukemia stem cell, should be examined. Several studies aimed at identifying cancer stem cells may provide a starting point for examining benzene-associated leukemia stem cells.
- Recent findings describing the bone marrow as a series of niches each of which plays a role in hematopoiesis are just beginning to be understood in terms of normal bone marrow function and must be evaluated with respect to the effects of hematotoxic agents.

The study of the mechanism of benzene toxicity and leukemogenesis has progressed slowly with about a half century intervening between descriptive initial studies of Santesson, Selling and Weiskotten and the early studies of Parke and Williams directed to understanding the mechanisms involved. Since then we have extended our appreciation of benzene metabolism. The next steps require that we take advantage of developments in studies of the bone marrow niches, stem cell biology, and the effects of perturbation of cell signaling if we are to delineate the mechanisms by which benzene causes leukemia. 
